# The Occurrence of Sexual Risk Behaviors and Its Association With Psychological Well-Being Among Kenyan Adolescents

**DOI:** 10.3389/frph.2021.659665

**Published:** 2021-07-21

**Authors:** Derrick Ssewanyana, Amina Abubakar, Adam Mabrouk, Vincent A. Kagonya, Carophine Nasambu, Judith Tumaini Dzombo, Vibian Angwenyi, Margaret Kabue, Gaia Scerif, Charles R. Newton

**Affiliations:** ^1^Neuroassement Group, Kenya Medical Research Institute (KEMRI)/Wellcome Trust Research Programme, Centre for Geographic Medicine Research, Kilifi, Kenya; ^2^Alliance for Human Development, Lunenfeld-Tanenbaum Research Institute, Toronto, ON, Canada; ^3^Department of Public Health, Pwani University, Kilifi, Kenya; ^4^Institute for Human Development, Aga Khan University, Nairobi, Kenya; ^5^Department of Experimental Psychology, University of Oxford, Oxford, United Kingdom; ^6^Department of Psychiatry, University of Oxford, Oxford, United Kingdom

**Keywords:** adolescents, sexual risk behavior, self-esteem, hope, psychosocial well-being, Kenya

## Abstract

**Objective:** Sexual risk behavior during adolescence is an important public health problem. Self-esteem and hopefulness are potentially important psychological factors that may play a role in the behavioral regulation mechanisms of adolescents. These factors are inadequately explored in sub-Saharan Africa. This study aimed at exploring patterns and associated factors for sexual risk behavior (SRB), self-esteem, and hopefulness among adolescents from a resource-poor setting in Kenya.

**Method:** A cross-sectional study conducted in 2019 among 296 adolescents (12–17 years old) from rural Kilifi (*n* = 133) and urban informal settings of Nairobi (*n* = 163) in Kenya. Participants completed the Kilifi Health Risk Behavior Questionnaire, Rosenberg self-esteem questionnaire, and Hope scale administered *via* computerized tablets. A binary outcome variable based on the experience of adolescents of at least one of the five forms of SRB: transactional sex, sexual violence, intergenerational sex, early sexual debut, and condom non-use was generated. Bi-variate analysis was conducted to summarize various social-demographic and psychosocial factors. A multivariable logistic regression model was fitted to investigate factors associated with the occurrence of SRB, self-esteem, and hopefulness among adolescents.

**Results:** About 13% of the participants had experienced a form of SRB, and among these, 36% reported co-occurrence of multiple forms of SRB. Adolescent SRB was largely characterized by having experienced sexual violence, as well as intergenerational and transactional sex. Higher scores of hopefulness were reported among adolescents who never experienced SRB (*P* = 0.03) at bivariate analysis level. However, both self-esteem and hopefulness were not significantly associated with the occurrence of SRB in the adjusted logistic regression analysis. Having depressive symptoms (Adj. OR = 3.8, 95% CI: 1.39–10.4), feeling unsafe in the neighborhood (Adj. OR = 3.4, 95% CI: 1.6–7.2), and being in higher compared with lower primary education level (Adj. OR = 0.3, 95% CI: 0.1–0.8) were statistically significantly associated with the occurrence of SRB.

**Conclusion:** Targeted reproductive health interventions, designed with the cognizance of structural and social drivers of adolescent SRB, are needed to concurrently tackle multiple forms of SRB. It is important to integrate mental health promotion within these interventions. More research is needed to understand the mechanisms and implications of self-esteem and hopefulness for adolescent sexual and reproductive health.

## Introduction

Sexual behaviors, such as transactional sex, early sex debut, intergenerational sex, inconsistent condom use, and sexual violence, that are associated with poorer health outcomes are often conceptualized as sexual risk behaviors (SRBs) (1). SRBs are reported as some of the major public health problems experienced by adolescents (10–19 years old) globally (2). In sub-Saharan Africa (SSA), SRB partly explains the unacceptably high incidence of HIV infection among adolescents and young women (25% in 2017) (3), and highest burden (19%) of teenage pregnancy compared to any other region (4). For example, a study conducted among school-going South African adolescents found that the odds for ever being pregnant were higher for girls who had two or more sexual partners (5). A cohort study among young women (15–24 years old) in Zimbabwe found that intergenerational relationships were significantly associated with concurrent relationships and an increase in HIV incidence. Moreover, the association between age disparity and HIV incidence did not change over time (6). Most countries with the lowest median age of sexual debut are in SSA (7). In Kenya, the median age for first sexual activity is 17 years, and a significant proportion of adolescents (26% of boys and 19% of girls) have initiated sex by the time they are 15 years of age (7).

Neurobiological developmental processes of the adolescent brain and various socio-ecological factors can contribute to risk-taking during adolescence. There is evidence that a heightened propensity for risk-taking during adolescence partly results from the curvilinear development of motivational behavior arising from the temporal gap between arousal of the socio-emotional system and the full maturation of the cognitive control system (8, 9). The early development of the socio-emotional system coincides with the onset of puberty (between 9 and 14 years); a stage during which impulsive sensation-seeking increases. It is explained that synaptic remodeling during puberty results in diminished inhibitory control, thus the heightened reward salience. Synaptic remodeling peaks by the age of 15 years, after which it begins to decline (9, 10). During late adolescence (i.e., 16 years and above), impulsive sensation-seeking begins to decline toward adulthood (i.e., until mid-20s), owing to the gradual development of the cognitive control system, which improves the self-regulation capacity of an adolescent (10). From a socio-ecological perspective, the engagement of adolescents in SRBs in SSA has been attributed to various factors that include individual level factors, such as male gender, level of religiosity (11–15), and substance use (16–19); interpersonal level factors such as peer influence (14, 20), household poverty, level of maternal education (11–15), and parenting behavior (17, 21–23); community level factors such as neighborhood factors, living arrangement and parenting behavior (17, 21–23), social cultural norms (24), and level of social support (17); and broader societal or public policy factors, for example, the level of accessibility to adolescent-friendly services coupled with the nature of the policy environment (25, 26) have been linked to the level of engagement of adolescents in SRBs in SSA.

Psychological factors, namely, self-esteem and hopefulness, have been associated with health risk behavior (27). It is important to understand how these individual psychological factors relate to the different SRBs, so as to design interventions that are most effective in protecting and promoting the sexual health and well-being of adolescents (28). Both hope and self-esteem are also concepts that carve out from the frameworks of reasoned action (29) and planned behavior (30) as well as positive psychology (31, 32). These approaches seem to agree that individuals tend to behave in a certain manner by considering one's attitude, values, beliefs, and perceived consequences of the behavior. An existing body of evidence suggests that self-esteem is a basic feature of mental health and has also been described as a buffer against negative influences, which actively promotes healthy functioning (33). A study found that stability of self-esteem was associated with self-regulatory styles, self-concept clarity, and goal-related affect (34). Although high self-esteem is generally believed to protect against unhealthy behavior (35), it is also reported that some people with high self-esteem engage in risk behavior, possibly because they employ a variety of self-serving cognitive strategies, such as over-estimating the prevalence of that behavior and by avoiding to think about the consequences, which in turn enables them to cope with the inconsistency between their risky behavior and their positive self-perception (36). Indeed, for SRB in adolescence, there have been some studies indicating protective effects of self-esteem (37–39), self-esteem as a risk for engagement in SRB (40), and the lack of significant link between self-esteem and SRB (41, 42). Indeed, the nature of the relationship between SRB and self-esteem remains unclear and warrants further investigation (43), especially in the SSA context where this research is still limited. Hope is conventionally understood as the perceived capability of a person to initiate pathways toward successful goal pursuit and to motivate themselves to use these pathways (44). The evidence indicates that adolescents with higher levels of hope experience a lower risk for internalizing behavior problems (45). It is posited that when operating from a hopeless perspective, adolescents are more likely to replace long-term goals, for example, maintaining a healthy lifestyle and good academic performance, with immediate benefits of engaging in risky behaviors (46). Unlike other forms of health risk behavior, such as substance use and injury-related behavior (47–51), the role played by hope in the sexual behavior of adolescents has been scantily researched (46, 52) and even much more scarcely researched within the SSA context. Furthermore, depression during adolescence is a common global health concern (53, 54). An extensive body of literature also shows that depression is associated with SRB during adolescence, whereby adolescents who report multiple SRB face an elevated risk of depression (55–57), which further underscores the need to investigate these factors simultaneously in studies on adolescent health.

Adolescents, especially in marginalized settings such as poor rural households and urban-poor informal settlements of Kenya, experience a myriad of psychosocial risk factors, which may impact immensely on their sexual reproductive health and rights. So far, there have been no reported studies within rural settings and urban informal settlements of Kenya that examine the important role of psychological factors of self-esteem and hopefulness on the occurrence of SRB during adolescence. Accordingly, the purpose of this study was to investigate patterns of SRB and whether the occurrence of SRB is associated with self-esteem and hopefulness among adolescents from rural and urban-poor settings in Kenya.

## Materials and Methods

### Study Design

A cross-sectional study was conducted among adolescents aged 12–17 years who are residents of a rural coastal setting in Kenya (Kilifi County) and two urban informal settlements in Nairobi (Kawangware and Mathare). The study was conducted between March and June 2019. Random stratified sampling, targeting 400 adolescents, that is, 200 from the rural setting and 200 from the urban informal settlements, was performed. In both Kilifi and Nairobi, extensive consultation with the involvement of community health assistants was conducted, and this helped to identify villages/clusters with high populations of adolescents. From each identified village, a community health volunteer worked closely with research assistants to recruit eligible study participants by visiting their households. In Kawangware, 116 adolescents assented; however, only 99 (54 females and 45 males) showed up for the assessments at The Aga Khan University's Institute for Human Development (IHD) field research office in Kawangware, Nairobi. In Mathare, out of the 87 adolescents who assented, 80 (49 females and 31 males) showed up for the assessments that were conducted in hired private rooms within a school complex. In Kilifi, of the 149 adolescents who assented, 135 (61 females and 74 males) showed up for the assessments at the Neuro-assessment Clinic at the Kenya Medical Research Institute (CGMRC-KEMRI). Both the adolescent and their accompanying caregivers/guardians took part in the assessments. Each adolescent was scheduled individually for a specific time slot, during which data collection was conducted in a private room at the assessment venue. Data were collected by trained research assistants using questionnaires administered *via* tablets. Specifically, for the health behavior questionnaires, the adolescents from Kilifi were administered the interviews *via* audio computer-assisted self-interview (ACASI), whereas the adolescents from Nairobi were administered a paper-based version of the questionnaires, which they self-filled using Swahili language. Since the health behavior questionnaire was previously adapted in the rural Kenya setting (58), cognitive interviews were conducted in Nairobi to ensure that the Swahili version of the questionnaire was contextualized with the sheng/slang spoken in the informal settlements. The caregiver of the adolescent only took part in responding to questions about the household socio-economic status and ascertaining socio-economic characteristics of date of birth and current education status of the child. The caregiver was asked these questions separately and was not permitted to be present while the adolescent was completing his or her assessment.

### Study Setting

Kilifi county comprises a predominantly rural population (61% by 2016) with adolescents (10–19 years old) accounting for about 1/4 (22%) of the population (59). Adolescents in Kilifi county experience various health and social challenges, as indicated by a more than national-level burden of teenage (15–19 years old) pregnancies (22%) (60), high levels of gender-based violence and school dropout (14, 61). Most (58%) residents in Kilifi live below the poverty line and close to 1/3 do not have formal education (62).

Both Kawangware and Mathare informal settlements are located in the capital city of Nairobi in Kenya. Kawangware is situated in Dagoretti sub-county, about 12 km from the central business district of Nairobi. Most of the residents of Dagoretti are low income earners who largely depend on the informal sector for a livelihood (63), and they are from a diverse background, such as immigrants and asylum-seekers from other countries and rural-to-urban Kenyan migrants. Limited access to improved sanitation and clean water, constrained access to health and social services, crowded low-quality housing, sanitation-related and communicable diseases (e.g., dysentery, malaria, HIV/AIDS, typhoid) are common in Dagorreti (63). However, the living conditions in Kawangware are better than those in other slums such as Kibera and Mathare.

Mathare is one of the oldest and the second biggest slum in Ruaraka sub-county of Nairobi with ~500,000 residents (64). It is about 5 km from the central business district of Nairobi. It is very densely populated, with most people living in shacks made of corrugated iron. The environment in Mathare is described as far from safe and with a general lack of basic amenities such as sanitation, waste management, drainage, electricity, running water, and proper road network (64).

### Participants

The study participants were adolescents aged between 12 and 17 years who reside in the aforementioned study sites and whose primary caregivers have provided written parental consent as well as them (adolescents) providing assent to take part in the study. As part of the exclusion criteria, emancipated minors, such as those who already had children or who were pregnant were not to be included in the study. All the participants were required to have adequate Swahili language comprehension and communication abilities, since the study tools were administered in this language. The ethics review for this study was obtained from the Research Ethics Committee of the Aga Khan University, Nairobi, Kenya and a research permit was granted by the National Commission for Science, Technology & Innovation (NACOSTI/P/19/48905/27951). Clearance to implement the research was obtained from the Directorate of Health Services and Education in Nairobi and Kilifi counties.

### Measures

In this study, the main outcome variable is the binary outcome variable based on the experience the adolescents of at least one of the five forms of sexual risk behavior. The main predictors are self-esteem, hopefulness, and depression. Other covariates include the social demographic variables of household socio-economic status; age, gender, current education level, of the adolescents, and their perception about feeling unsafe in their neighborhood. These are described in detail below:

#### Sexual Risk Behavior (SRB)

Five elements of SRB were assessed using items from the Kilifi Health Risk Behavior Questionnaire (KRIBE-Q). The KRIBE-Q was previously adapted and validated as a measure for the health behavior of adolescents in rural coastal Kenya (58). It showed a good spread of responses across options, a very good test–retest reliability (overall Gwet AC1 = 0.82) and was feasible to administer *via* audio-computer assisted self-interview (ACASI). The KRIBE-Q captures eight behavioral categories, among which the items on sexual behaviors were found to possess acceptable test-retest reliability (Gwet's AC1 = 0.94) (58). The specific elements were: *early sexual debut* (having ever had sexual intercourse at 13 years or less); *sexual violence* (having ever been physically forced or coerced to have sexual intercourse when the person did not want or consent to it); transactional sex during the past 12 months (either paying or getting paid or giving/receiving gifts or any other goods in exchange for sexual intercourse in the last 12 months); intergenerational sex during the past 12 months (having had sexual intercourse in the past 12 months with a sexual partner who was 10 years older); and condom non-use during most recent sexual intercourse.

#### Self-Esteem

The Rosenberg self-esteem questionnaire, which is a 10-item self-reported measure of global esteem, was administered to the adolescents to assess this outcome. The measure is scored to range from 0 to 30 with higher scores indicating higher self-esteem and vice versa. This tool has been widely used among adolescent subpopulation in Kenya (65, 66), and has been validated in various regions such as East Africa (67). In this study, this measure was found to have a good internal consistency (alpha = 0.71).

#### Hopefulness

The hope scale (68) is a 12-item scale, of which eight items yield hope and four are filler items. Some studies have shown this scale to have a high internal consistency (e.g., alpha = 0.82) when used among adolescents and youths (69). The scale had an acceptable internal consistency (alpha = 0.61) in this study. Higher total scores on the hope scale indicate higher levels of hopefulness and vice versa.

Depressive symptoms of the participants over the past 2 weeks were assessed using a Swahili version of the Patient Health Questionnaire (PHQ-9). The Swahili version of the PHQ-9 has been found to have acceptable internal consistency, test–retest reliability, and good discriminant validity within the Kenyan setting (70). Participants with a PHQ-9 total score ranging from 0 to 9 are classified as non-depressed, while those with 10 and above total scores are classified as depressed (71).

##### Social Demographic and Other Variables

Household socio-economic status was assessed by asking the caretakers of the adolescents about their household possessions using an assets index, which has been previously utilized in adolescent studies in a similar study setting (72).

The age, gender, current education level, such as an item on whether they had ever repeated any grades, of the adolescents were captured and ascertained in the presence of their caretakers plus from available records. Such resources included the most recent academic report card of the adolescent and a birth certificate or any other formal document indicating the date of birth of the adolescent. An additional item drawn from the Kilifi health risk behavior questionnaire was used to ask participants if they felt unsafe in their neighborhood (home or school or on their way from school or any other place).

### Statistical Analysis

We generated a binary outcome variable based on the experience of the adolescents of at least one of the five forms of sexual risk behavior, namely, early sexual debut (having ever had sexual intercourse at 13 years or less), sexual violence, transactional sex during the past 12 months, intergenerational sex during the past 12 months, and condom non-use during most recent sexual intercourse. We used descriptive statistics of proportions, means, and standard deviations to summarize the various socio-demographic characteristics of the participants who experienced and did not experience SRB. We also conducted bi-variate analyses, such as *t-*test and chi-square test (or Fisher's exact test instead of chi-square test when individual cells had fewer than 10 observations) to assess for statistical differences between the two groups (i.e., those who experienced and did not experience SRB). We also summarized a breakdown of the multiplicity (combination) of the forms of SRB among those who had experienced SRB.

Logistic regression was conducted to investigate whether the occurrence of SRB is associated with self-esteem and hopefulness among adolescents. The independent variables included in the multivariate analysis had a *P-*value of 0.25 or less (*P* ≤ 0.25) in the initial bi-variate analyses (73). We performed multiple imputation for data missing at random (74), for some independent variables. A *P-*value of 0.05 or less (*P* ≤ 0.05) was considered as the cutoff mark for statistical significance.

## Results

Of the 314 adolescents who participated in the study, we successfully appended data on SRB and other study relevant variables of 296 adolescents. We did not come across any emancipated minors during the recruitment. Of the 296 participants, 55% were from urban informal settlements, and the overall mean age was 14 years (SD = 1.6). We did not find statistical significant differences in age between the urban and rural adolescents. The proportion of females was significantly greater (58%) in the urban sample, whereas the rural sample had a significantly higher proportion of males (57%) [*P* < 0.01]. All the adolescents attended school. There was a greater proportion of participants in upper primary and secondary education level among the rural adolescent (91%) compared with those from the urban setting (61%) [*P* < 0.001]. Overall, 44 (15%) of the participants reported engagement in some form of sexual behavior, and most of them (61%) were males. Most (43%) of those who engaged in some form of sexual behavior were secondary school students, whereas 30 and 27% were in upper and lower primary education levels, respectively. Of the 44 participants who engaged in some form of sexual behavior, 11% did not experience any of the five forms of SRB, 57% reported engagement in only a form of SRB, equal proportion (14% for each) engaged in two and three forms of SRB, and 4% engaged in four forms of SRB. In total, the adolescents who did not experience any of the five forms of SRB together with those who were abstinent (i.e., never experienced any SRB) comprised 87% of the overall sample in this study.

### Sociodemographic and Psychological Characteristics of Adolescents Across SRB Groups

Table 1 summarizes the sociodemographic and psychological characteristics across the adolescent SRB groups. Group comparisons showed there were no significant differences in residence (*P* = 0.86), age (*P* = 0.94), gender (*P* = 0.12), class repetition (*P* = 0.65), and household socioeconomic status (*P* = 0.26) between the adolescents who experienced SRB and those that did not. We found that the adolescents who had not experienced SRB had significantly higher hopefulness scores than those who had experienced SRB (71.6 vs. 67.8, *P* = 0.03). However, these differences were not present for the self-esteem component (*P* = 0.41). A significantly higher proportion of the adolescents had depression (31 vs. 11.9%, *P* = 0.006) and expressed feelings of insecurity (i.e., fearfulness) (43.6 vs. 19.8%, *P* = 0.001) among those who had experienced SRB in comparison with those who had not.

**Table 1:** Socio-demographic and psychosocial characteristics of the adolescents summarized by experience of sexual risk behavior.

**Variables**	**Overall sample (*****N*** **= 296) n (%)**	**Never experienced any SRB (*****N*** **= 257), n (%)**	**Experienced one or more forms of SRB (*****N*** **= 39), n (%)**	***P*** **-value**
Study site				0.86
Rural	133 (45.0)	116 (45.1)	17 (43.6)	
Urban	163 (55.0)	141 (54.9)	22 (56.4)	
Age (mean, SD) years	14.1 (±1.6)	14.1 (±1.6)	14.0 (±2.0)	0.94
Gender				0.12
Males	143 (48.6)	120 (46.9)	23 (60.5)	
Females	151 (51.4)	136 (53.1)	15 (39.5)	
Education level				**0.01**
Lower primary	65 (22.1)	53 (20.8)	12 (30.8)	
Upper primary	149 (50.7)	138 (54.1)	11 (28.2)	
Secondary	80 (27.2)	64 (25.1)	16 (41.0)	
Class repetition				
Yes	100 (34.0)	88 (34.5)	12 (30.8)	0.65
Household assets (mean, SD)	2.5 (±2.5)	2.5 (±1.6)	2.9 (±1.7)	0.26
Self-esteem scores (mean, SD)	19.6 (±3.9)	19.6 (±3.9)	19.1 (±3.7)	0.41
Hopefulness scores (mean, SD)	71.1 (±10.4)	71.6 (±10.4)	67.8 (±9.8)	**0.03**
Depression^*^				**0.006**
Yes	34 (14.2)	25 (11.9)	9 (31.0)	
Had feelings of insecurity in his/her neighborhood				
Feeling unsafe	68 (22.9)	51 (19.8)	17 (43.6)	**0.001**

* *Assessment for depression (n = 236); chi-square or Fisher's exact test (for categorical variables). Bolded results indicate that there is statistically significant difference [i.e. statistical significance level > 0.05]*.

### Occurrence of SRB

A total of 39 (13%) adolescents reported having experienced some form of SRB. Of those who had experienced SRB, about 1/3 (36%) reported multiple forms of SRB, with the highest being four out of the total five forms of SRB among 5% (see Table 2). Noteworthy is that sexual violence (having ever been physically forced to have sexual intercourse when the person did not want or consent to it) was the most experienced form of SRB both among those who reported one or multiple forms of SRB. This was also coupled with a high occurrence of transactional sex, highlighting a high degree of age and economic asymmetry in SRB.

**Table 2:** Breakdown of the forms of experienced sexual risk behaviors (SRB) among the study participants reporting SRB (*N* = 39).

**Forms of SRB**	**N (%)**	**Sub-total N(%)**
1. **SRB**		25 (64.1)
Sexual violence (lifetime)	9 (36.0)	
Condom non-use (most recent sex)	5 (20.0)	
Early sexual debut	5 (20.0)	
Intergenerational sex (past 12 months)	4 (16.0)	
Transactional sex (past 12 months)	2 (8.0)	
2. **SRB**		6 (15.4)
Early sexual debut + Condom non-use	2 (33.2)	
Transactional sex + Intergenerational sex	1 (16.7)	
Transactional sex + Sexual violence	1 (16.7)	
Sexual violence + Intergenerational sex	1 (16.7)	
Sexual violence + Condom non-use	1 (16.7)	
3. **SRB**		6 (15.4)
Sexual violence + Transactional sex + Intergenerational sex	2 (33.3)	
Sexual violence + Early sexual debut + Intergenerational sex	2 (33.3)	
Early sexual debut + Intergenerational sex + Transactional sex	1 (16.7)	
Sexual violence + Early sexual debut + Condom non-use	1 (16.7)	
4. **SRBs**		2 (5.1)
Sexual violence + Intergenerational sex + Transactional sex + Condom non-use	2 (100.0)	

### Factors Associated With Experiencing Sexual Risk Behaviors

Results from the multivariate analysis indicate that both psychological factors of hopefulness [Adj. OR = 0.97, 95% CI (0.94, 1.01), *P* = 0.16] and self-esteem [Adj. OR = 0.98, 95% CI (0.89, 1.09), *P* = 0.77] were not statistically significantly associated with the experience of the adolescents of SRB while adjusting for the factors of depression, education level, gender, and feelings about safety in the neighborhood (see Table 3). The findings show that both depression and the feeling of insecurity in the neighborhood are positively associated with experiencing SRB while holding the other factors constant. The odds of experiencing SRB were more than 3.5 times higher among those with depression compared with the non-depressed adolescents [Adj. OR = 3.8, 95% CI (1.39–10.4), *P* = 0.009], and they were three times higher among those who felt unsafe compared with their counterparts who felt safe in their neighborhood [Adj. OR = 3.4, 95% CI (1.56–7.17), *P* = 0.002]. The odds of experiencing SRB were significantly [Adj. OR = 0.30, 95% CI (0.11–.81), *P* = 0.018] lower among the adolescents in upper primary compared with those in lower primary education level.

**Table 3:** Results from multivariate logistic regression showing factors associated with the experience of the adolescents of sexual risk behavior (*N* = 292).

**Variables**	**Odds ratio (95% CI)**	***P*** **-value**
Hopefulness	0.97 (0.94, 1.01)	0.16
Self-esteem	0.98 (0.89, 1.09)	0.77
Has depressive symptoms		**0.009**
No (Reference)		
Yes	3.80 (1.39, 10.4)	
Education Level		
Lower primary (Reference)		
Upper primary	0.30 (0.11, 0.81)	**0.018**
Secondary	1.11 (0.43, 2.85)	0.83
Gender		0.08
Females (Reference)		
Males	1.93 (0.92, 4.08)	
Feeling unsafe in neighborhood		**0.002**
Feeling safe (Reference)		
Feelingunsafe	3.35 (1.56, 7.17)	

*Bolded results indicate that there is statistically significant difference [i.e. statistical significance level > 0.05]*.

## Discussion

In this study, we found that most of the adolescents had not experienced any of the five forms of SRB. Given their young age, it is not surprising that they were not sexually active. Of note, a small proportion (about 13%) who experienced SRB is unacceptably high among this young subpopulation. Moreover, 1/3 of those adolescents experienced multiple or co-occurring forms of SRB, which may suggest the presence of multi-faceted sources of risk and vulnerability to sub-optimal adolescent sexual and reproductive health within this group. Noteworthy is that the co-occurrence of health risk behaviors (including multiple forms of SRB) has been previously reported among adolescents in rural Kenya (75). Another study conducted among adolescents from informal settlements of Nairobi reported the co-occurrence of early sex debut and involvement with multiple sexual partners among 14% of participants (76). This finding highlights the need for well-targeted sexual health interventions to concurrently address multiple sources of risk and specific needs of the adolescents within distinct vulnerability profiles.

The findings from this study indicate that risky sexual experiences among the adolescents in this study setting are characterized by a high occurrence of sexual violence, coupled with intergenerational and transactional sex. We found that a large proportion (32%, i.e., 14 adolescents) of the total number of sexually active adolescents had engaged in intergenerational sex and about 43% (i.e., 6 adolescents) of those who engaged in intergenerational sex also experienced transactional sex. Moreover, 1/4 (i.e., 11 adolescents) of the sexually active adolescents indicated that they did not use condoms during their most recent sex. The findings on non-condom use practices, exploitive practices such as transactional sex and intergenerational sex, coupled with experiences of adolescents of coerced sex are corroborated by other studies on Kenyan adolescents in both rural (14, 77) and urban-poor settings (78–81). For instance, a study on 1,365 orphaned adolescents in rural western Kenya found that overall, 9% had experienced sexual coercion and an equal proportion had ever engaged in transactional sex (77). Another study involving 1,754 adolescents from two informal settlements in Nairobi reported that 10% of the sexually active females had engaged in intergenerational sex (an additional 10% had older partners but were not sure of the age difference), and 18% of sexually active females were not at all willing to have sex during their first debut. In the same study, close to 62% (both males and females) of those who had ever had sexual intercourse indicated that they did not use any form of contraception or used a traditional method during their first sexual intercourse (78). As the sample is relatively small, we anticipate some variations in the proportions of adolescents reporting SRB if a much larger sample is utilized. Nonetheless, the findings importantly highlight that among a considerably large portion of young adolescents in this study setting, sexual intercourse often involves little control over safe sex practices, for example, condom use, but also high vulnerability to sexual exploitation and violence (82). These findings stress the need to strengthen the implementation of and the update of existing laws and policies addressing sexual and gender-based violence in addition to other forms of injustices experienced by adolescents within the Kenyan setting (83). Researchers have called for the need to design interventions with cognizance of the structural and social drivers, which underlie transactional and intergenerational sex, as well as the recognition that sometimes girls in transactional relationships may not consider themselves as being exploited (84).

Another key finding was that self-esteem and hopefulness were not statistically significant predictors of SRB once adjusted for depression, education level, gender, and feeling unsafe. Nonetheless, based on the results from the bi-variate analysis, we found a significantly higher score of hopefulness among those who had not experienced SRB, suggesting some potential protective effects of hopefulness against SRB. The result showing that adolescents who engage in SRB report greater hopelessness than those who do not is corroborated by findings from studies conducted among young women from rural South Africa (52) and adolescents from low-income urban communities (46, 85). While this effect was not statistically significant according to the *P-*value in the multi-variate analysis model, its confidence interval in the model is relatively narrow (0.94–1.01), which may suggest that the study is possibly underpowered to detect an independent direct effect of hopefulness. Similar to the findings on self-esteem, studies on adolescents from Uganda (41) and the United States (42) did not find a significant association between self-esteem and sexual behavior of adolescents. It is plausible that most of the adolescents from both of the rural and urban-poor study settings experience shared psychosocial risks, such as household poverty and suboptimal living conditions, which potentially impact their self-esteem and hopefulness in comparable magnitude. This is also supported by the absence of differences in household socioeconomic status between the adolescents from the rural and urban-poor settings in the analysis. Indeed, poverty and income disparities have been shown to impact self-esteem and hopefulness among adolescents (52, 86, 87). However, in contrast to the findings, studies conducted on adolescents in Nigeria (37), USA (28, 38), and the Netherlands (39) showed that higher self-esteem was associated with lower engagement in SRB. There are also other studies that have found higher self-esteem among adolescents with greater engagement in SRB (40). It is also plausible that hopefulness is a mediating or intermediate factor that could potentially explain mechanisms through which other factors lead to SRB. Such investigation was beyond the scope of this study; however, from exploratory analysis, we observed that in adjusted multivariate analyses, the variable “hopefulness” had remained statistically significantly associated with SRB until the introduction of the variable “feeling unsafe in the neighborhood” into the multivariate model. As there was not a confounding effect or interaction, it is likely that the effects of feeling unsafe in the neighborhood mask those of hopefulness in explaining SRB of adolescents. This assumption is partly supported by the findings from an adolescent cohort study in the United Kingdom, which suggest that perceived neighborhood safety and emotional problems of adolescents influence each other partly because of experiences of risky behavior (88). The findings of this study indicate that the nature of the relationship between SRB and the psychological aspects of self-esteem and hopefulness during adolescence remains unclear and therefore requires more rigorous research. We recommend the use of larger sample sizes in future studies. Higher-powered studies could potentially identify the effect of hopefulness at a statistically significant level. However, it is highly likely that the effect size of hopefulness would still be smaller compared with other predictors such as depression and fearfulness.

The finding of this study indicates that lower education attainment among adolescents who experienced SRB compared with those who had not experienced SRB is corroborated by findings from other adolescent health studies within the Kenyan context (89, 90). It is also reported that while most young adolescents (i.e., those who are 0–14 years old) have not initiated sexual intercourse, some are already exploring intimate relationships without adequate information to make informed decisions about their sexual and reproductive health (91). Higher educational attainment may be one way through which adolescents learn more about their developing bodies and obtain more exposure to sexual health information, which could possibly lessen the vulnerability to multiple SRBs. Therefore, keeping adolescents in school is beneficial. Moreover, early adolescence presents an opportune window of time to provide comprehensive sexuality education, which improves informed decisions about their sexual health (91).

Another key finding was that both depression and feeling unsafe (fearfulness) were associated with the occurrence of SRB. First, the findings on an elevated level of depression and/or feeling unsafe in the neighborhood among adolescents who are vulnerable to health risk behavior (including SRB) are corroborated by similar findings within rural (66, 72) and urban-poor (92) settings of Kenya. Overall, an extensive body of literature indicates that adolescents who experience risky sexual behavior (such as violent and exploitive experiences) experience elevated levels of mental illness (56, 93, 94), as well as negative emotional outcomes such as low self-esteem and diminished positive outlook. Mental illness is also shown to negatively impact on the level of self-esteem and positive outlook of adolescents (95, 96). When left unaddressed, depression and internalizing problems can escalate into life-threatening public health consequences such as self-harm, suicide, serious psychotic conditions, and other mental health-related mortalities (97–99). The findings stress the need for integration of mental health screening, treatment, and prevention services into routine adolescent health promotion programs within this study setting. Addressing the mental health of adolescents in these settings requires more holistic approaches that take into proper consideration various social determinants of health and sources of daily stressors, for example, household poverty, insecurity, crime, and marginalization (100).

## Strengths and Limitations

This study crucially builds onto a currently under-explored topic on the role of the psychological factors of hope and self-esteem in adolescent sexual and reproductive health, especially in the sub-Saharan context where adolescent health outcomes remain suboptimal amidst scanty adolescent specific data (101). The focus on adolescents from rural and urban poor settings of Kenya is also timely, as adolescents from such settings tend to be ignored and yet are likely to face disproportionately greater vulnerability to suboptimal sexual health outcomes as compared with their peers from other settings. The findings should, however, be interpreted with caution, since a relatively small sample size of 296 was studied. Although comparable with the sample sizes in a number of studies on SRB of adolescents (28, 37, 102), the sample may present some limitations in generalization and detection of some statistical differences. All measures of SRB, self-esteem, and hopefulness were self-reported, and there is a possibility that some level of social desirability bias and recall bias may have been introduced. However, self-reported assessment of health behavior and psychological well-being of adolescents is conventionally utilized and considered reliable (103). Besides, we used measures that have been previously psychometrically tested and pre-piloted within the study setting (58, 70). The study being cross-sectional by design meant that it was not possible to ascertain the direction of the associations explored in this one.

## Conclusion

About one-tenth of the adolescents from the rural and urban-poor study context have experienced SRB, among whom 36% report multiple forms of SRB. Interventions addressing SRB of adolescents are likely to be most effective when they are age-appropriate and address multiple drivers of SRB, to concurrently tackle multiple forms of SRB. Noteworthy is that sexual experiences of adolescents were largely marked by sex coercion, age, and economic asymmetry, suggesting the urgent need to design interventions with cognizance of the structural and social drivers of transactional and intergenerational sex, and to prioritize more stringent implementation of laws and policies addressing sexual and gender-based violence plus other forms of injustices faced by adolescents. A need to incorporate mental healthcare and prevention components in adolescent sexual and reproductive health programs within this study setting is crucial. Future studies, preferably with larger sample sizes and longitudinal in design, are needed to further explore the patterns and trajectories of SRB in addition to the role played by psychological factors of self-esteem and hopefulness during adolescence, especially among poor marginalized settings.

## Data Availability Statement

The raw data supporting the conclusions of this article will be made available by the authors, without undue reservation.

## Ethics Statement

The studies involving human participants were reviewed and approved by Research Ethics Committee of the Aga Khan University, Nairobi, Kenya. Written informed consent to participate in this study was provided by the participants' legal guardian/next of kin.

## Author Contributions

CNe, AA, and GS contributed to conception and design of the study. CNa and VA organized the database. DS performed the statistical analysis. DS and AM wrote the first draft of the manuscript. MK, VA, JD, CNa, and VK contributed important details in the methodology sections of the manuscript. All authors contributed to manuscript revision, read, and approved the submitted version.

## Conflict of Interest

The authors declare that the research was conducted in the absence of any commercial or financial relationships that could be construed as a potential conflict of interest.
